# Impact of the coronavirus disease 2019 (COVID-19) pandemic on antimicrobial stewardship programs in Colorado hospitals

**DOI:** 10.1017/ash.2022.24

**Published:** 2022-10-21

**Authors:** Caleb L. Matteson, Christopher A. Czaja, Matthew P. Kronman, Sonja Ziniel, Sarah K. Parker, Daniel S. Dodson

**Affiliations:** 1 Section of Pediatric Infectious Diseases, Department of Pediatrics, Children’s Hospital Colorado, University of Colorado School of Medicine, Aurora, Colorado; 2 Colorado Department of Public Health and Environment, Denver, Colorado; 3 Division of Pediatric Infectious Diseases, Department of Pediatrics, Seattle Children’s Hospital, University of Washington, Seattle, Washington; 4 Section of Pediatric Infectious Diseases, Department of Pediatrics, University of California Davis, Sacramento, California

## Abstract

Using a mixed-methods approach, we assessed the effect of the coronavirus disease 2019 (COVID-19) pandemic on antimicrobial stewardship programs (ASPs) in Colorado hospitals. ASP leaders reported decreased time and resources, reduced rigor of stewardship interventions, inability to complete new initiatives, and interpersonal challenges. Stewardship activities may be threatened during times of acute resource pressure.

The coronavirus disease 2019 (COVID-19) pandemic placed an acute strain on hospital resources including those dedicated to antimicrobial stewardship programs (ASPs).^1,2^ Given the importance of ASPs in improving patient outcomes and combating antimicrobial resistance,^3^ we characterized the effect of the pandemic on ASPs in Colorado.

## Methods

We conducted a mixed-methods evaluation including a survey and semistructured interviews with the overarching objective of characterizing and assisting Colorado ASPs. The study was not considered human-subjects research by the University of Colorado Internal Review Board.

### Recruitment

We targeted all 103 acute-care and critical-access hospitals in Colorado. We sent e-mails to hospital stewardship leaders using a list maintained by the Colorado Department of Public Health and Environment (CDPHE) and advertising through the Colorado Hospitals Association. In these communications, we described the study aims and emphasized voluntary participation. Data were collected from October 2020 to May 2021.

### Survey

The survey was pilot tested prior to implementation. Questions included categorical descriptions of time dedicated to stewardship and assessed the effect of the pandemic on prospective audit and feedback (PAF) and prior authorization, including whether interventions were continued and changes in the number of patients, or days per week interventions were performed.

### Interview

The interview guide was created and pilot tested with guidance from qualitative experts at the Adult and Child Consortium for Health Outcome Research and Delivery Science in Aurora, Colorado. Discussion of the COVID-19 pandemic was prompted by the question, “How has COVID-19 affected your stewardship practices?” Such discussion often arose spontaneously in other areas of the interview, which were conducted virtually and lasted 30–60 minutes.

### Analysis

Only complete surveys were included. Survey data were exported to Stata version 16 software (StataCorp, College Station, TX) for analysis.^4^ Interviews were recorded, transcribed, and uploaded to NVivo 12 software (QSR International, Burlington, MA) for inductive thematic analysis.^5^ Transcripts were provided to each participating hospital with no subsequent feedback given. Interviews were independently coded and are discussed to ensure consistency. Themes were identified as they arose from the coding.^6^ The codebook was reviewed by all authors.^7^

## Results

In total, 41 hospitals completed the survey for a 40% response rate, including 25 acute-care hospitals (ACHs; 35%) and 16 critical-access hospitals (CAHs; 50%). Among the respondents, 24 hospital ASP leaders were interviewed, including 14 ACHs and 9 CAHs. Thematic saturation was reached prior to interviewing all 24 hospitals, but interviews were continued to facilitate collaboration. Hospital size ranged from 11 to 698 beds (median, 52 beds; interquartile range, 25–219).

### Survey results

Of the 41 responding hospitals, 39 (95%) quantified total time for ASP. Among them, 11 hospitals (28%) reported decreased time compared to 3 (8%) that reported increased time during the pandemic compared with the prepandemic era. Of the 26 hospitals with physician ASP leaders, 25 (96%) quantified physician leader time for ASP. Among them, 6 hospitals (24%) reported decreased time and 4 hospitals (16%) reported increased time as a result of the pandemic. Of the 27 hospitals with pharmacist ASP leaders, 18 (67%) quantified pharmacist leader time for ASP. Among these, 9 hospitals (50%) reported decreased time and 2 hospitals (11%) reported increased time during the pandemic.

**Fig. 1.**
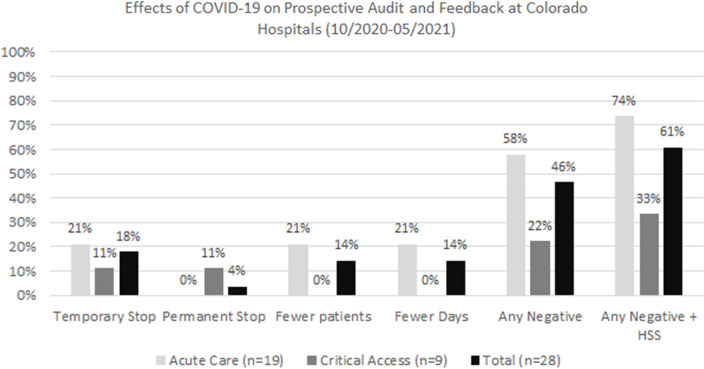
Effects of the COVID-19 pandemic on prospective audit and feedback. Any negative effect refers to any temporary or permanent stop of PAF or use on fewer patients or fewer days. Any negative effect including HSS refers to any of the previous negative effects plus hospitals stopping handshake stewardship (temporarily or permanently). Note. HSS, handshake stewardship; PAF, prospective audit and feedback.

Of 28 programs conducting PAF prior to the pandemic, 5 (18%) temporarily stopped, 1 (4.2%) stopped and had not restarted, 4 (14%) did PAF on fewer days during the pandemic, and 4 (14%) did PAF on fewer patients during the pandemic (Fig. 1). In total, 17 programs (46%) had at least 1 of these negative effects. No hospitals reported performing PAF on more patients or more days during the pandemic. Of the 24 programs implementing handshake stewardship prior to the pandemic (PAF with in-person recommendations), 8 programs (33%) temporarily stopped handshake stewardship and 2 additional programs (8.3%) stopped and had not restarted handshake stewardship at the time of survey completion. Overall, 17 PAF programs (61%) reported at least 1 negative effect of the pandemic when stopping handshake stewardship was included. The 14 prior-authorization programs were similarly evaluated and minimally affected; only 1 program temporarily stopped their prior authorization protocol.

### Interview results

In total, 24 ASPs were interviewed, and themes were stratified by negative and positive effects of the pandemic on stewardship. The impact of the COVID-19 pandemic on ASPs across Colorado was overwhelmingly negative. Overall, 20 interviews (83%) included a negative theme, compared to only 4 (16.7%) interviews mentioning positive themes. The most common negative themes included interpersonal challenges, decreased time and personnel, and decreased rigor and progress of ASPs (Table 1).

**Table 1.** Summary of the Negative Effects of COVID on ASPs with Supporting Quotations—Colorado Hospitals, October 2020–May 2021

Theme	Subtheme	Affected HospitalsNo. (%)	Supporting Quotation
Interpersonal challenges	Communication	12 (50)	“Since that time [COVID], chat has become much more highly used. It’s just so much more—I like that personal interaction. I really liked having that personal interaction. I would go to the physician’s lounge, and sometimes we still will do rounds in the physician’s lounge. I can get people there and talk to people. Now, with COVID, obviously, there’s a lot less interaction on the floors. People are so busy that I tend to send the messages and let them get back to me when they have a chance.” Record 10
	Burnout	3 (13)	“We’ve had a lot of—the providers are pretty frustrated with just the town’s perception of the coronavirus and practices right now, so people are not really doing what they should. Employees are contracting it too, so I think there’s just a lot of frustration and burnout, fatigue with that.” Record 24
Stewardship time and personnel loss	Time loss	18 (75)	“There’s no time to do anything… I moved in in February. I have a dry erase board that I filled out the day I moved in with all my goals for the year. It literally says, “Coronavirus prep. Get guidelines up in the ED, and then ongoing communication.” It’s the dry erase board that time forgot.” Record 5
	Role conflict	14 (58)	“For 6 months, we were—it [COVID] required a significant amount of our resources to the extent that the last thing that really we were thinkin’ about was stewardship, quite honestly.” Record 36 “We don’t have the time, and multiple responsibilities, and sometimes things other than stewardship take priority.” Record 6
	Employee turnover	8 (33)	“I had one [an assistant]. I miss her every day. Actually, I should have put that in the big COVID changes. Her funding was cut with COVID.” Record 3
Decreased progress in stewardship programs	Tracking/Reporting	7 (29)	“In an ideal world, it’s [tracking] once a day. With COVID, it’s kind of backed down to maybe once a week, first thing like the carbapenem reviews. For bacteremia’s it tends to be more once a day.” Record 23
	Action	7 (29)	“Our pharmacy team was pulled from the floors during our surge. We were still on site but working from a library space. Our ability to really have that handshake stewardship was a bit limited.” Record 28 “Then we then also provided direct feedback, most recently kind of in a more similar academic fashion where we would go to the floor round with some of the teams, give them feedback about their antibiotic use and step-downs, so it was a multifactorial project. This was all before COVID.” Record 6
	Antibiotic prescription dilemmas	7 (29)	“Our biggest struggle in COVID is trying to prevent broad spectrum empiric therapy for pneumonia and trying to get de-escalation to occur at a reasonable time.” Record 23 “Just simply even that, just trying to look at the COVID patients. Are they on the right treatments?” Record 10
	Education	2 (8.7)	“In my view, it’s [rounding] much it’s much less effective, much more frustrating. A lot less teaching and education. Not any of those really quick little side lectures that we had been doing.” Record 3
	General stagnation	13 (54)	“We didn’t get reports generated. We didn’t have any kind of in-person meetings. All of our goals got set on the backburner. It affected everything. Not just antibiotics stewardship. We’ve not moved forward I don’t feel like for nine months. We’ve just been stuck in COVID hell.” Record 5

*Interpersonal challenges.* Changes to communication structures during the pandemic were discussed in 12 interviews (50%). An increased reliance on online chat functions and telecommunication was noted in 8 interviews (33%), and cessation of in-person communication was reported in 7 interviews (29%). The shift away from in-person communication was not seen as productive for ASPs; it compromised interpersonal communication and working relationships among ASP members. Handshake stewardship efforts were especially disrupted with transition from in-person to remote rounding. Burnout and anxiety were mentioned in 3 interviews (13%), including frustration with community perceptions of the pandemic.

*Decreased time and personnel.* Decreased time for ASP due to COVID-19–related activities was discussed in 18 interviews (75%). Moreover, 14 interviewees (58%) described role conflict in their program, being “forced to wear multiple hats,” as stewardship was neglected in favor of COVID-19–related activities, including COVID patient management, therapeutics, vaccine clinics, infection control procedures, and staying up-to-date with new developments. Moreover, 8 interviewees (33%) mentioned that employee turnover related to the pandemic negatively affected stewardship. Budget cuts and increased hospital census was discussed in 4 interviews (17%).

*Decreased rigor and progress of ASP.* ASP teams were often unable to round as effectively during the pandemic compared to prior according to 7 interviews (29%), and handshake stewardship, a keystone of many effective stewardship programs,^8^ was disrupted by the shift to remote communication, according to 2 interviews (8.3%). Additionally, COVID-19–related antibiotic dilemmas were common, including perceived increases in broad-spectrum antibiotic use in 4 interviews (17%) and difficulties navigating the treatment of SARS-CoV-2 and the role of antibiotics in 3 interviews (13%). Negative impacts on tracking and reporting antibiotic use were described in 7 interviews (29%), primarily related to a lack of time available. A sense of programmatic stagnation was discussed in 13 interviews (54%), again largely related to scarcity of time allocated to ASP. Such stagnation was accompanied by fewer ASP meetings in 5 interviews (21%) and was compounded by weakened relations between ASP providers, which was mentioned in 4 interviews (17%). These interviewees also mentioned that relationships were weakened by the shift to online communication and by stress and burnout. Finally, 2 interviewees (8.3%) discussed an inability to further their ASPs through education.

*Positive themes.* Although they were the exception, positive effects of the pandemic on ASPs were reported. Most notably, 3 programs (12.5%) noted stronger connections and resource sharing with public health officials and regional hospitals (eg, consultations, incorporating data from outside the facility, and resource sharing).

## Discussion

The shift of healthcare resources toward pandemic-related activities diminished rigorous antimicrobial stewardship in Colorado, primarily due to siphoned time and resources from stewardship to COVID-19–related activities, and this shift compromised ASP communication structures. The negative impact on ASP was compounded by factors related directly to the pandemic including reduced in-person interactions, employee burnout, and uncertainty concerning the role of antibiotics in COVID-19 therapy. As the COVID-19 pandemic continues, and new national, local, and hospital-specific challenges are always on the horizon, dedicated attention must be given to antimicrobial stewardship to combat the developing threat of antimicrobial resistance and to optimize patient outcomes.
